# Comparative Evaluation of Dexmedetomidine and Magnesium Sulphate on Propofol Consumption, Haemodynamics and Postoperative Recovery in Spine Surgery: A Prospective, Randomized, Placebo Controlled, Double-blind Study

**DOI:** 10.15171/apb.2016.012

**Published:** 2016-03-17

**Authors:** Vinit K. Srivastava, Abhishek Mishra, Sanjay Agrawal, Sanjay Kumar, Sunil Sharma, Raj Kumar

**Affiliations:** ^1^ Apollo Hospitals Bilaspur, Chhattisgarh, India.; ^2^ Himalayan Institute of Medical Sciences, Dehradun, India.; ^3^ Sanjay Gandhi Postgraduate Institute of Medical Sciences, Lucknow, India.

**Keywords:** Dexmedetomidine, Magnesium sulfate, Hemodynamics, Spine surgery

## Abstract

***Purpose:*** Dexmedetomidine and magnesium sulfate have been used in anesthesia as adjuvant to provide hemodynamic stability and anesthetic agents sparing effect. We compared these effects of dexmedetomidine and magnesium sulfate in spine surgeries.

***Methods:*** Ninety patients were randomly assigned to three groups. Group D received dexmedetomidine loading dose 1 µg/kg over a period of 15 minutes and maintenance 0.5 µg/kg/h throughout the surgery. Group M received magnesium sulfate loading dose 50 mg/kg over a period of 15 minutes and maintenance 15 mg/kg/h throughout the surgery. Group C received same volume of normal saline. Heart rate (HR) and blood pressure values were recorded at various intervals. The induction and maintenance doses of anesthetics and recovery parameters were also recorded.

***Results:*** Heart rate in group D and group M were significantly decreased (p<0.05) during the whole intraoperative period compared to preoperative values. There was a significant difference in HR values between group C, D and M, during the whole intraoperaive period (p<0.05). Blood pressure values were statistically significantly lower in the group D and group M compared to group C after intubation and all time observations of surgery (p<0.05). Both drugs reduced the anesthetic agent’s requirement during surgery. However, the recovery parameters were statistically significant increase with magnesium sulphate compared to dexmedetomidine and control groups.

***Conclusion:*** Dexmedetomidine is more effective than magnesium sulfate for maintaining the hemodynamic stability in spine surgeries. Both these drugs also reduce the requirements of anesthetic agents. Recovery from dexmedetomidine is as rapid as control group compared to magnesium sulfate.

## Introduction

Spine surgery poses challenges to anesthesiologists due to its specific needs of stable hemodynamics, relatively dry operative field, the need for intraoperative somatosensory or motor evoked potential as well as perioperative position. These factors warrant the use of medications to maintain adequate depth of anesthesia as well as specific need of the surgery.

The use of perioperative magnesium sulfate,^1,2^ dexmedetomidine,^3,4^ beta blocker (esmolol),^5^ opioids,^6^ gabapentin, pregabalin,^7^ and clonidine^8^ have been reported to provide beneficial effects during general anesthesia, however with varying success rate.

Magnesium sulfate (MgSO_4_) is a non-competitive N-methyl- D-aspartate (NMDA) receptor antagonist with antinociceptive effects.^9,10^ Numerous clinical investigations have demonstrated that Mg infusion during general anesthesia reduced anesthetic requirement and postoperative analgesic consumption.^1,2^

Perioperative use of alpha-2 adrenoceptor agonists decreases sympathetic tone, attenuates the stress responses to anesthesia and surgery, sedation and postoperative analgesia. Dexmedetomidine is a highly specific alpha-2 adrenergic receptor agonist.

Few studies in the literature demonstrate the effectiveness of dexmedetomidine and magnesium sulfate individually for providing intraoperative hemodynamic stability, decrease anesthetic requirements and improved postoperative recovery in general anesthesia.^1,2,3,4^ This present prospective, randomized, placebo-controlled, double blind study was designed to evaluate and compare the pharmacological effects of the use of dexmedetomidine and magnesium sulfate on propofol consumption, hemodynamics and postoperative recovery in patients undergoing spine surgery under general anesthesia.

## Materials and Methods

This prospective, randomized, placebo-controlled study was conducted after approval from the Institutional Ethics Committee and written informed consent from the patients. The study was registered at Clinical Trials.gov www.ctri.nic.in (ref: CTRI/2013/08/003939).

A total of 106 patients 20-60 years of age, ASA physical status I or II, of either sex, and scheduled for elective spine surgery under general anesthesia were included in this study. Patients with a history of preoperative neuromuscular disease, hepatic, renal, endocrinal, hematological disorder or cardiovascular dysfunction, any degree of heart block, BMI>30 kg/m^2^, patients receiving magnesium supplementation, drugs known to have a significant interaction with NMDAs, chronic use of opioids and current treatment with a β-blocker or calcium channel blocker were excluded from the study. The 90 patients were randomly allocated to three groups of 30 each with the help of a computer generated table of random numbers.

Group D – Dexmedetomidine loading dose 1 µg/kg before induction over a period of 15 min and maintenance 0.5 µg/kg/h throughout the surgery.

Group M – Magnesium sulfate loading dose 50 mg/kg before induction over a period of 15 min and maintenance 15 mg/kg/h throughout the surgery.

Group C (Control group) - The same volume of normal saline was administered

In the operating room, preloading was done with 10 ml/kg of normal saline. Routine monitoring of electrocardiography (ECG), pulse oximetry and blood pressure were established before starting study drug. Radial artery cannulation was performed under local anesthesia. Electrodes were applied to the patient’s forehead for monitoring the bispectral index (BIS) of the electroencephalogram. Neuromuscular transmission was monitored using train-of-four (TOF) supramaximal stimulations (2 Hz, 50 mA; TOF Watch SX, Organon Ltd, Dublin, Ireland).

All the drugs were prepared by an independent anesthesiologist not involved in the study, in identical syringes and infused with infusion pump (Perfusor Compact, B Braun, Germany). The loading doses of the drugs were administered 20 min prior to induction. Induction of anesthesia in all the groups was started 5 minutes after termination of the loading dose of study drug.

Anesthesia was induced with inj. midazolam 0.03 mg/kg, inj. fentanyl 1 µg/kg and inj. propofol 10 mg every 5 seconds until the BIS below 60 followed by vecuronium 0.1 mg/kg body weight and intubation completed with appropriate size cuffed endotracheal tube. Anesthesia was maintained with oxygen: nitrous oxide (O_2_:N_2_O; 40:60), and propofol infusion to achieve a target BIS between 40 and 60. Ventilation was adjusted to maintain an end-tidal carbon dioxide (ETCO2) between 35 and 40 mmHg. Propofol consumption was noted every hour. Presence of hypertension (mean arterial pressure > 20% from baseline) or tachycardia with maintaining BIS between 40 and 60 was treated with a bolus dose of fentanyl 0.5 µg/kg. TOF supramaximal neuromuscular stimulation (2 Hz, 50 mA) was measured at 5 min intervals to the end of surgery. Vecuronium (0.015 mg/kg) was administered when TOF count exceeded 2 until 10 min before the end of surgery. After skin closure, all infusion drugs were stopped and neuromuscular blockade was reversed with inj. neostigmine (40 μg/kg) and inj. glycopyrolate (10 μg/kg) when T4/T1 ratio reached 75% or higher followed by tracheal extubation. The time elapsed between stoppage of propofol infusion and a BIS value of 80 was considered as the recovery time. The time of cessation of anesthetics to tracheal extubation, response to verbal commands and orientation were also recorded.

Heart rate (HR), systolic blood pressure (SBP), diastolic blood pressure (DBP), mean arterial pressure (MAP) and BIS were recorded at preoperative, after study drug administration, after induction, after intubation, intraoperative period at 20 min intervals, after surgery and after extubation.

Any hypotension (MAP <30% baseline) was managed with a fluid bolus of 200 ml of normal saline. If hypotension did not respond to fluid administration, inj. mephentermine 5 mg i.v. was administered. If hypotension did not respond to 2 repeat doses of mephentermine then other means were sought as per the need. Any incidence of bradycardia (HR <50/min) was treated with inj. atropine 0.6 mg i.v.

The sample size was calculated by power analysis, using a two-sample *t* test, with a two sided type I error of 5% (α=0.05) and power at 80.37 (α=0.19). Therefore 25 patients in each group were needed to detect an intergroup difference of at least 20% in blood pressure and heart rate. We enrolled 30 patients in each group to account for potential dropouts or protocol violations.

Statistical analysis was performed using the Graph pad prism 6.0 statistical software. Patient characteristic data were analyzed with one way analysis of variance (ANOVA) for continuous variables and Chi square test for categorical variables. Intergroup comparison of hemodynamic parameters were done with one way analysis of variance (ANOVA), followed by an unpaired t test. Repeated measure analysis of variance (ANOVA) with the *post hoc* Tukey test was used to compare means for hemodynamic variables in intragroup comparison to baseline parameters. A P value of <0.05 was considered statistically significant.

## Results

**Figure 1:**
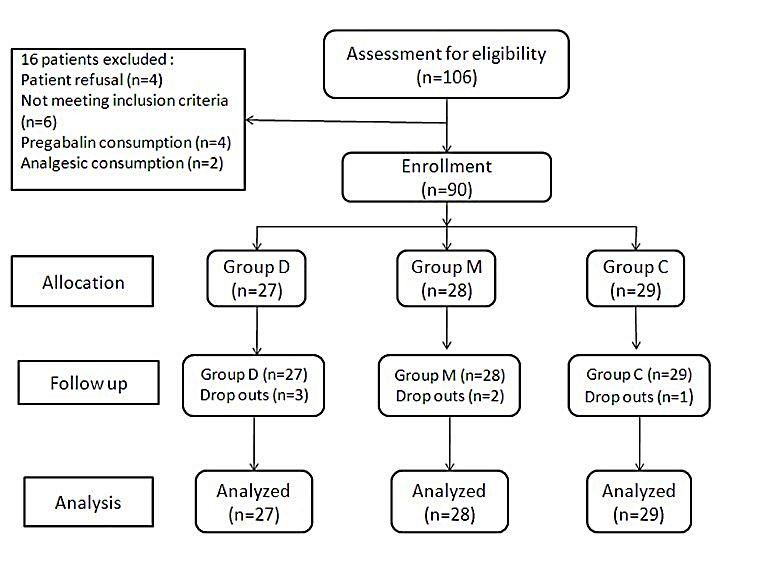
Study Design

A total of one hundred six patients were assessed for eligibility, out of which ninety patients were included in the study after randomization and eighty four patients (93.33%) completed the study (Figure 1). Sixteen patients were excluded in this study on account of patient’s refusal (four patients), not meeting inclusion criteria (six patients), pregabalin consumption (four patients) and analgesic consumption (two patients). Six patients were not included in this study on account of ventricular ectopic (one patient in group M), hypotension in group D (two patients), bradycardia (one patient in group D) and blood loss (two patients, one in group M and one in group C) which require blood transfusion. Their data has been included in the comparison of demographic profile; however, they were not subjected to further statistical analysis.

There was no significant difference amongst the groups with regard to demographic variables (P>0.05) (Table 1). Propofol induction dose and maintenance dose were significantly lower in the group D and group M than in the group C (P<0.05). Vecuronium and fentanyl maintenance doses were also significantly lower in the group D and group M than in the group C (P<0.05) (Table 2).

**Table 1:** Demographic data

-	**Group C** **(n=30)**	**Group D** **(n=30)**	**Group M** **(n=30)**	**P Value**
**Mean age (yrs)**	46.57 ±8.73	45.93±9.19	48.30±7.70	0.543
**Weight (Kg)**	62.27±10.19	60.37 ±7.20	64.70 ±8.12	0.153
**Male/Female**	16/14	18/12	17/13	0.873
**Spine surgery** **Cervical/Lumbar**	10/20	8/22	11/19	0.713
**Duration of surgery (hrs)**	2.73±0.62	2.62±0.59	2.41±0.48	0.087

Data are presented as either mean values ± SD or by absolute numbers

**Figure 2:**
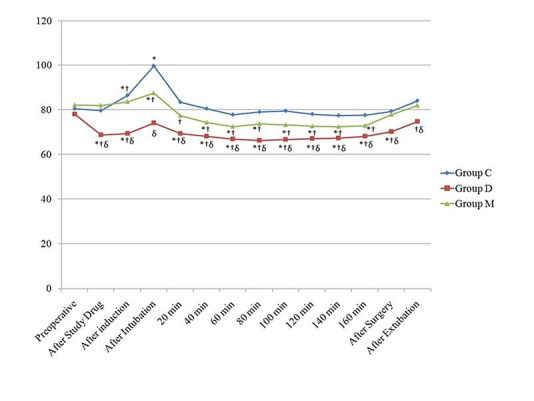
Changes in the heart rate (HR) observed in the three groups during the study period. *P˂0.05 within group (vs baseline value), † P < 0.05 compared with group C, δ P < 0.05 group D vs group M.

There was no significant difference in preoperative hemodynamic parameters between the groups. After administration of the study drugs, there was a significant decrease in heart rate in group D (p<0.05). After induction, there was no change in HR in group M only. There was no significant increase in HR in group D after intubation (p>0.05). HR in group D and group M were significantly decreased (p<0.05) during the whole intraoperative period except 20 min in group M, however, this decrease was not seen in group C, compared to preoperative values. There was no significant change in HR after surgery and extubation in all groups except in group D after surgery (p<0.05). There was a significant difference in HR values between group C, D and M, during the whole intraoperaive period (p<0.05) (Figure 2).

**Table 2:** Induction dose of propofol and maintenance dose of propofol, vecuronium and fentanyl

**Variable**	**Group C** **(n=29)**	**Group D** **(n=27)**	**Group M** **(n=28)**	**P value**		
				**C vs D**	**C vs M**	**D vs M**
**Propofol Induction dose (mg)**	106.03±20.93	74.07±12.41	95.35±12.54	<0.001	<0.05	<0.001
**Propofol Maintenance dose (mg/hr)**	213.41±47.82	155.60±34.63	180.34±29.63	<0.001	<0.01	<0.05
**Vecuronium Maintenance dose (mg/hr)**	2.21±0.82	1.82±0.48	1.40±0.40	<0.05	<0.001	<0.05
**Fentanyl Maintenance dose (µg/hr)**	44.38±10.40	25.07±8.03	34.93±8.44	<0.001	<0.001	<0.001

Data are presented as mean values ± SD

**Figure 3:**
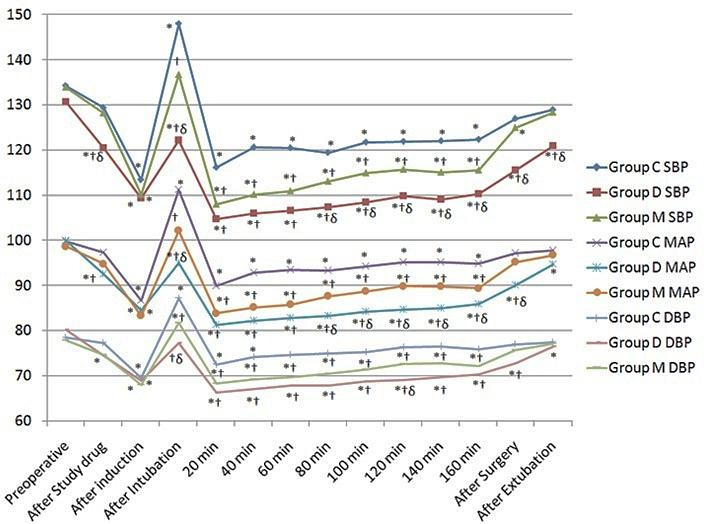
Changes in the blood pressure (SBP, DBP and MAP) observed in the three groups during the study period. *P˂0.05 within group (vs baseline value), † P < 0.05 compared with group C, δ P < 0.05 group D vs group M.

SBP and DBP values were statistically significantly lower in the group D after study drug administration. Intubation caused significant increased in SBP and DBP values in group C only, compared to preoperative value. SBP values were statistically significantly lower in all three groups at all time observations of surgery compared to preoperative values. In group C there was no statistically significant change in DBP values after 40 min to all times of observations. These values were statistically significantly lower in the group D and group M after intubation and all time observations of surgery, when compared with the group C (p<0.05). There was no significant difference of SBP and DBP values between group C and group M after surgery (p>0.05). After extubation, there was no significant difference in SBP values only between group C and group M (Figure 3).

MAP values were statistically significantly lower in the group D and group M comparative to group C after intubation and all time observations of surgery (p<0.05). There was a significant decrease in MAP in all groups, compared to preoperative values at all time intervals of surgery (p<0.05). There was no significant difference in MAP after surgery between the group C and M (p=0.237). MAP values difference were more when compared the group D with group C (p<0.001), than group M with group C (p<0.05) (Figure 3).

Extubation time, response to verbal command and orientation time were significantly delayed in group M when compared with group C and D (p<0.05) (Table 3). In group M, serum magnesium levels increased from the pre-operative level 1.59±0.17 mg/dl to 2.10±0.26 mg/dl (postoperative value).

Hypotension was observed in only two patients (6.66%) in group D, which responded to administration of 2 doses of mephentermine 5 mg i.v. and one patient (3.33%) in group D developed bradycardia, which required atropine 0.6 mg i.v. One patient in the group M developed ventricular ectopic during the intraoperative period (serum Mg 3.0 mg/dl) without change in HR and blood pressure.

## Discussion

In spine surgery hemodynamic stability is most important as sudden intraoperative hypertensive episodes causes intraoperative bleeding and it impairs quality of vision of the surgical field leading to an increased rate of complications. Therefore, improving the visibility of the surgical field by reducing bleeding during spine surgery is an important issue for anesthesiologists. In spine surgery, agents providing controlled hypotension and total intravenous anesthesia have emerged with the purpose of surgical field clarity. Therefore, we tested the superiority of two agents, magnesium sulfate and dexmedetomidine, against each other for maintaining hemodynamic stability and consumption of anesthetic agents.

Our study demonstrated that perioperative use of dexmedetomidine and magnesium sulfate is associated with hemodynamic stability as well as reduced anesthetic consumption. The degree of decrease of perioperative consumption of propofol and fentanyl was 20% more with dexmedetomidine compared to magnesium.

**Table 3:** Recovery parameter

**Variable**	**Group C**	**Group D**	**Group M**	**P Value**		
				**C vs D**	**C vs M**	**D vs M**
**Extubation time (min.)**	10.78±2.98	11.19±3.55	13.39±3.65	0.633	<0.05	<0.05
**Response to verbal command (min.)**	9.82±2.59	10.34±3.11	12.68±3.29	0.496	<0.01	<0.05
**Orientation time (min)**	11.81±2.86	12.57±3.65	14.68±3.19	0.386	<0.01	<0.05

Data are presented as mean values ± SD

Dexmedetomidine was associated with a higher incidence of hypotension and bradycardia and this effect is dose related. We chose to study a dose of 1µg/kg over 15 min duration and maintenance 0.5 µg/kg/h and this dose are supported by various studies.^3,4,11^ Magnesium sulfate is also safe to use as an adjuvant. There have been cases of magnesium toxicity leading to cardiac arrest and death.^12^ However, magnesium toxicity begins at serum concentrations of 2.5–5.0 mmol/litre, which is much higher than the highest level in Group M in this study. Goral N, et al^13^ also noted that toxic level of serum magnesium concentration is not reached even after using magnesium sulfate in the dose of bolus (50mg/kg) and continuous infusion (20 mg/kg/hr). In the present study, we administered magnesium sulfate bolus dose (50 mg/kg), and the maintenance dose (15 mg/kg/hr) based on previous studies.^1,2,14^

In the present study dexmedetomidine group achieved a 20% more decrease in propofol and fentanyl requirements comparison of magnesium group with the control group. This reduction in dexmedetomidine group is due to the hypnotic, sedative, analgesic and anesthetic sparing effect of dexmedetomidine. The interaction of α2-adrenoreceptors and opioids lead to decrease in the dose of fentanyl. The α2 adrenoceptors have an effect on the spinal cord, especially α2A and α2C as well as modulating the descending noradrenergic pathways leading to 30% to 50% reduction in the requirements of opioids.^15^ Our study is in accordance with other studies^16,17^ Magnesium could modulate anesthesia by several mechanisms. The mechanism of analgesic effect of magnesium is due to interference with calcium channels and antagonism of the NMDA receptor in the CNS. The first possibility is based on the observation that calcium channel blockers have an antinociceptive action and enhance opiate analgesia in patients with cancer chronically treated with morphine. Magnesium blocks NMDA-induced currents in a voltage-dependent manner by blocking the receptor channel effects,^9^ second possible explanation for the analgesic action of magnesium. Telci et al^18^ and Choi et al^19^ demonstrated significant reductions in infusion rates of propofol and opioids, titrated to maintain BIS between 45 and 60 by using a magnesium infusion throughout the operations. These results are similar to our study.

It is well known that magnesium sulfate prolongs and potentiates neuromuscular block by non-depolarizing neuromuscular blocking agents. Consistent with previous studies^1,20,21^ our study also showed that lower vecuronium requirements with magnesium use. Fuchs-Buder et al^22^ reported that an intravenous infusion of magnesium at 40 mg/kg significantly potentiated the neuromuscular blockade of vecuronium, and plasma magnesium concentrations increased significantly from a baseline after the magnesium sulfate infusion, but no symptoms of muscle weakness were observed in any patients. In addition, Baraka and Yazigi^23^ found no clinical or electromyographic signs of muscle weakness, even at slightly higher plasma magnesium concentrations (1.7–2.5 mmol/L).

In the present study, both the study drugs (dexmedetomidine and magnesium sulfate) have better hemodynamic stability with minimum fluctuations during whole intraoperative period. Dexmedetomidine group has been associated with a greater decrease in HR, in part because of the sympatholytic effects of this drug, but also because of a vagal mimetic effect. From postintubation to the end of surgery, MAP remained at a significantly lower level in dexmedetomidine and magnesium sulfate treated subjects than in controls; this probably indicates that the analgesic properties of dexmedetomidine and magnesium sulfate reduce sympathetic stimulation. Magnesium produces vasodilatation by directly acting on the blood vessels and by interfering with a wide range of vasoconstrictor substances. In addition to its direct effects on the vessel wall, raised serum magnesium levels may also reduce peripheral vascular tone by a number of other mechanisms, including sympathetic blockade and inhibition of catecholamine release. Dexmedetomidine also is known to decrease sympathetic outflow and circulating catecholamine levels and would therefore be expected to cause a decrease of MAP similar to those of magnesium. The hypotension and bradycardia that occurred in the dexmedetomidine group were predictable from the known properties of α2 agonists, and have been confirmed from previous studies.^24,25^

Similar to our results, Ryu, et al^21^ showed lower trends of hemodynamic parameters with attenuation of stress response to intubation and surgery in the magnesium group. Our study is also supported by previous other studies of magnesium sulfate use as an adjuvant. Soliman RN, et al^26^ used the same dose of dexmedetomidine in craniotomy patients and showed that it maintained the hemodynamic stability, reduced sevoflurane and fentanyl requirements and improved significantly the outcomes. Turgat N, et al^6^ used dexmedetomidine 0.6 μg/kg as bolus before induction and 0.2 μg/kg/hr by infusion during spine surgery and found that MAP values were better controlled in dexmedetomidine treated patients except after intubation comparison to fentanyl group and we felt this result may be due to low dose of dexmedetomidine. In our study, we used different doses of dexmedetomidine in spine patients and found that MAP is better controlled at all points of the observations.

The major problem with dexmedetomidine is its hemodynamic effects, as the drug often causes hypotension and bradycardia. In our study, hypotension (6.66%) and bradycardia (3.33%) were also noted and it was also found in other studies.^27,28^

In the present study, patients received magnesium sulfate were associated with significantly longer recovery time, this effect was also reported in previous studies when they added magnesium sulfate to anesthetic regimen.^8,18,22^ Few studies also observed that there was a no significant increase in the recovery parameter in magnesium sulfate treated patients when monitored through TOF.^20,21^ We also monitored TOF but we have given vecuronium until 10 min. prior to the end of surgery, because some of our patients are in the prone position and this causes hemodynamic derangements during change of position.

There are some limitations of this study. First, the use of nitrous oxide might confound the interpretation of BIS. Second, we determine the serum magnesium concentration before and after surgery in only group M due to cost factor. So the relationship between serum magnesium and analgesic effect could not be evaluated. Third, we used fixed doses of study drugs. Therefore, further experiments on the different doses of study to minimize adverse effects of drug are needed.

## Conclusion

Dexmedetomidine and magnesium sulfate led to a significant reduction of propofol, fentanyl, vecuronium requirements and maintained the haemodynamic stability during intubation and surgery. Dexmedetomidine was associated with bradycardia and hypotension and magnesium sulfate caused a delay in recovery.

## Acknowledgments

The authors thank Mr. Teku Ram Kashyap, Mr Jogendra Kumar and the surgical staff for all their assistance and suggestions during the current study.

## Ethical Issues

Not applicable.

## Conflict of Interest

The authors report no conflicts of interest.
